# A DNA-Methylated Sight on Autoimmune Inflammation Network across RA, pSS, and SLE

**DOI:** 10.1155/2018/4390789

**Published:** 2018-08-12

**Authors:** Xingqiang Wang, Dongyun Lei, Jie Ding, Shuang Liu, Li Tao, Fan Zhang, Jiangyun Peng, Jian Xu

**Affiliations:** ^1^Department of Rheumatology and Immunology, First Affiliated Hospital of Kunming Medical University, Kunming, Yunnan 650032, China; ^2^Department of Rheumatology and Immunology, The No. 1 Affiliated Hospital of Yunnan University of Traditional Chinese Medicine, Kunming, Yunnan 650021, China; ^3^Department of Dermatology, Tianjin Academy of Traditional Chinese Medicine Affiliated Hospital, Tianjin 300120, China; ^4^Department of Liver Diseases, The Third People's Hospital of Kunming City, Kunming, Yunnan 650041, China

## Abstract

Methylation variabilities of inflammatory cytokines play important roles in the development of systemic lupus erythematosus (SLE), rheumatoid arthritis (RA), and primary Sjögren's syndrome (pSS). With heightened focus on personalized and precise medicine, it is necessary to compare and contrast the difference and similarity of cytokine methylation status between the 3 most classic autoimmune diseases (AIDs). In this study, we integrated 5 Cytokine-Chips from genome-wide DNA methylation datasets of the 3 kind of AIDs, delta-beta value was calculated for intergroup difference, and comprehensive bioinformatics analyses of cytokine genes with aberrant methylations were performed. 125 shared differential methylation variabilities (DMVs) were identified. There were 102 shared DMVs with similar methylation status; 3 hypomethylated differential methylation regions (DMRs) across the AIDs were found, and all 3 DMRs were hypomethylated. DMRs (AZU1, LTBR, and RTEL1) were likely to serve as activator in the inflammatory process. Particularly, AZU1 and LTBR with hypomethylated TSS and first exon located in the promoter regions were able to trigger inflammation signaling cascades and play critical roles in autoimmune tautology. Moreover, functional epigenetic module (FEM) algorithm showed that different inflammatory networks are involved in different AIDs; 5 hotspots were identified as biologically plausible pathways in inducing or perpetuating of inflammation which are epigenetically deregulated in AIDs. We concluded methylation variabilities among the same cytokines can greatly impact the perpetuation of inflammatory process or signal pathway of AIDs. Differentiating the cytokine methylation status will serve as valuable resource for researchers alike to gain better understanding of the epigenetic mechanisms of the three AIDs. Even more importantly, better understanding of cytokine methylation variability existing between the three classic AIDs will aid in identification of potential epigenetic biomarkers and therapeutic targets. This trial is registered with ChiCTR-INR-16010290, a clinical trial for the treatment of rheumatoid arthritis with Warming yang and Smoothening Meridians.

## 1. Introduction

Autoimmune diseases (AIDs) can result in the loss of immune tolerance to self-antigens and involvement of specific organ or multiple organs. AIDs affect ~8% of individuals worldwide; annual incidence reported is still increasing due to escalating environment pollution and improved clinical diagnoses. The chronic nature of AID signifies a heavy burden on economy and patients' quality of life [1, 2]. AIDs' etiology is typically multifactorial. Aside from genetics and environment factors, imbalance of inflammatory mediator including cytokines and chemokines plays a critical role in the pathogenesis of AIDs. Cytokines can form a complex cytokine regulatory network which affect many important physiological functions of the human body. Imbalance of the anti- or proinflammatory cytokines can precipitate the development and perpetuation of autoimmune processes and ultimately leads to progression of AID.

DNA methylation of CpG dinucleotides is a well-studied epigenetic mechanism; it interacts with other cellular regulatory components to regulate levels of gene expression instead of by changing DNA sequence. Variability of gene expression regulation may partly explain why a proportion of genetically susceptible individuals do not manifest disease symptoms. Epigenetic mechanism would be critical in understanding the link between environmental factors, genetic influences, development, and progression of diseases [3, 4]. Cytokine-related genes have been demonstrated to be susceptible to epigenetic regulation, such as essential genes for T-effector pathways. Variability in DNA methylation levels contributes to recruitment and expression balance of inflammatory cytokines and to the development of AIDs. Identification and quantification of methylation levels in AIDs would serve as potential epigenetic biomarkers. Regulating methylation level would also be a potential drug target for the development of therapies.

AIDs share similar pathophysiology, subphenotypes, and genetic factors but have their own unique clinical manifestations in different patients [2]. In classic AIDs, systemic lupus erythematosus (SLE), rheumatoid arthritis (RA), and primary Sjögren's syndrome (pSS), arthritis is a common manifestation, but in RA patients, arthritis can lead to bone erosion, which is not the case in most SLE and pSS patients [5]. Several recent studies have assessed the genome-wide DNA methylation profiling of SLE, RA, and pSS; studies found that different clinical manifestations were likely to be closely related to different methylation levels of inflammatory cytokines. However, the specific differences and relationships of the classic representatives of AIDs are not fully studied.

In this study, we searched, merged, and selected a list of cytokine-relevant genes which involved chemokines, interferons, interleukins, lymphokines, transforming growth factors, and tumor necrosis factors from *Gene* database and employed a computational strategy by integrating multiple Cytokine-Chip from genome-wide DNA methylation datasets of SLE, RA, and pSS. This study provides basis for future research in exploring genetic association between SLE, RA, pSS, and inflammation.

## 2. Materials and Methods

**Figure 1:**
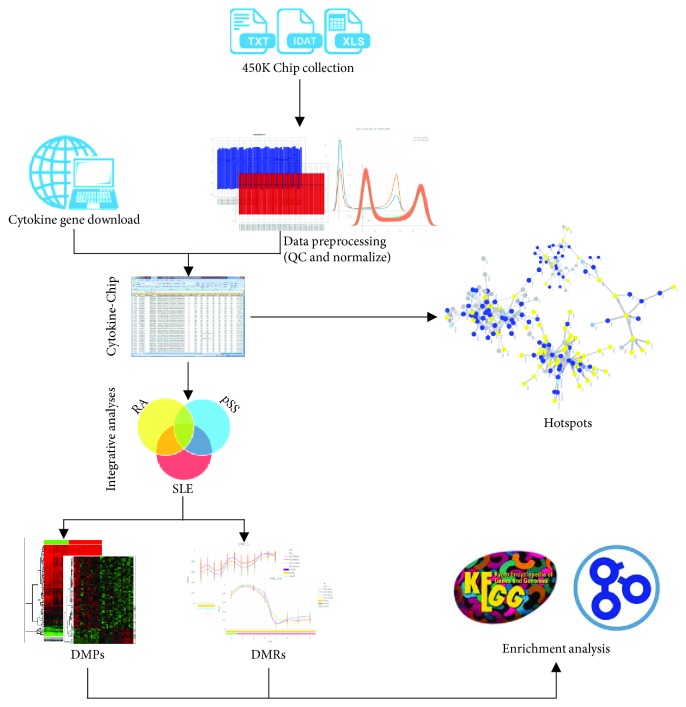
Workflow of methodology applied in this study. Step 1: the collection of methylated chip datasets of RA, pSS, and SLE and the conduction of quality control and normalization. Step 2: the generation of Cytokine-Chips. Step 3: the investigation of cytokine-cytokine interactome hotspots. Step 4: the integrative analysis for identifying differences and similarities on DNA methylation status across the 3 AIDs. Step 5: the GO and KEGG enrichment analysis of cytokine genes with aberrant methylation. QC, quality control; DMPs, differential methylated positions; DMRs, differential methylation regions; 450K Chip: Illumina Infinium HumanMethylation450 BeadChip.

The workflow of this study was depicted in Figure 1.

### 2.1. Datasets

All methylated chip datasets used in this study were retrieved from the Gene Expression Omnibus (GEO) and the European Bioinformatics Institute (EBI). Then we filtered the datasets via inclusion criteria as follows. (i) The organism was human being and samples used to isolate DNA belonged to peripheral blood; (ii) DNA methylation status was detected by Illumina HumanMethylation450 BeadChip (450K Chip); (iii) raw data (.IDAT or .TXT format) of enrolled dataset was available online.

### 2.2. Study Design and Subject Information

This study was composed by three kinds of datasets of autoimmune diseases, namely, dataset RA, dataset pSS, and dataset SLE. Each of three all has two groups (case and control groups). Dataset RA was sourced from GSE42861 [6] and consisted of 354 patients with RA, aged 51.15 ± 12.05, and 335 healthy individuals (age: 52.76 ± 11.45). This dataset was a Swedish population-based case-control study, and the cases were diagnosed by the American College of Rheumatology (ACR) classification criteria for rheumatoid arthritis. Dataset pSS was sourced from GSE75679 [7] and consisted of 48 patients with pSS (age: 56.54 ± 13.72) who fulfilled the American-European Consensus Group (AECG) 2002 classification criteria for pSS [8]. The controls in this dataset were 50 healthy individuals (age: 55.04 ± 7.76) who were matched with database of GSE42861 using random sample methods. Dataset SLE was a collection of GSE59250 [9], GSE65097 [10], and GSE82218 [11]. And the diagnosis of SLE all was performed according to ACR classification criteria for lupus [12]. Details of subjects' characteristics enrolled in this study are shown in Additional File 1 Table S1. Finally, 684 patients and 580 controls with autoimmune diseases were included in our study and there is no statistical difference in gender and age distributions in patients with autoimmune diseases and controls, respectively (Figure S1 in Additional File 1).

### 2.3. Data Preprocessing

For each chip datasets, quality control and data normalization were performed using the minfi or ChAMP packages for R [13, 14]. And the following quality criteria were performed: (i) failed probes' ratio per sample more than 10% or probes with <3% beads less than 5% of sample per probe must be discard; (ii) all probes overlapped SNP loci from 1000 Genomes Project must be removed [15]; (iii) all multihit probes and probes located in Y chromosome were also discarded. After quality checking, data was preprocessed using beta-mixture quantile dilation normalization strategy [16].

### 2.4. Generation of Cytokine-Chips

In order to identify the similarities and differences of three autoimmune diseases in inflammation, we produced a Cytokine-Chip by annotation methods. Firstly, we searched, merged, and selected a list of cytokine genes which included chemokines, interferons, interleukins, lymphokines, transforming growth factor, and tumor necrosis factors from *Gene* database (https://www.ncbi.nlm.nih.gov/gene/). There are 656 cytokine-relevant genes in this gene list, and descriptions and characteristics of these genes are shown in Additional File 2 and in Additional File 1 Figure S2, respectively. Then we found out all methylated probes in 450K Chip according to the gene list above. So these found probes formed a new beadchip that measured the DNA methylation of inflammation-relevant genes. There were 9948 probes in it, and these probes covered 3393 GpG islands (34.11%), 684 shelves (6.88%), and 2254 shores (22.66%), which are mainly located on chromosomes 1, 2, 6, and 19 (Additional File 1 Figure S2B and S2C).

### 2.5. Analysis of DMVs of Each Datasets

The previous analysis has given 5 Cytokine-Chips for 5 chip datasets. Because dataset RA and dataset pSS corresponded to one Cytokine-Chip, but dataset SLE corresponded to three, we performed the standard pipeline for RA and pSS, and SLE also conducted meta-analysis based on the standard pipeline.

The standard pipeline to identify DMPs was accomplished using dmpFinder algorithm in minfi package [13, 17]. We selected significant DMPs between cases and controls after applying Benjamini-Hochberg method (FDR < 0.05). But for dataset pSS, we used a stringent method (the *P* value is corrected by Bonferroni method and adj. *P* < 0.05, |Δ*β*| ≥ 0.01) due to its controls from stochastic sampling. The meta-analysis was completed by Meta package [18], and the *P* values of meta-analysis were corrected through setting Benjamini-Hochberg-adjusted threshold of 0.05. DMRs, genomic regions with abnormal methylation levels, were defined as clusters of probes having at least 2 consecutive probes within 1 kb distance in which methylations were significantly enriched or depleted between two groups. We identified DMRs (≥5 neighboring positions) and mapped to human genome (hg19) using DMRcate method with R package “ChAMP.” Meanwhile, we also performed stringent threshold (adj. *P* < 0.05,|Δ*β*| ≥ 0.01) to reduce the potential impact of extreme *β* value on methylation difference and to identify potentially biologically important CpG sites.

### 2.6. Identifying Differences and Similarities of DNA Methylation

The Δ*β* was used to assess differences and similarities of DNA methylation among three disorders. The Δ*β* was calculated by the following formula:

(1) $$\begin{array}{c} \Delta \beta =\beta_{case}–\beta_{control}, \end{array}$$

where *β*_case_ is the mean of beta value in the case group, and *β*_control_ is the mean of beta value in the control group. The methylation status was also defined by Δ*β*. When Δ*β* of a site was negative, the site presented a status with hypomethylation, and we labeled it “HypoM”; when Δ*β* was positive, the site shown a higher methylation level, and we labeled it “HyperM”; when Δ*β* equaled to zero, it did not have a flag due to the same methylation level between the cases and controls. So we were able to identify the differences and similarities of DNA methylation by the flags. The label of one of the three diseases was identical, indicating that the three diseases had similar methylation patterns, namely, similarity, and other situations represented difference.

In this identification, null hypothesis was that there was no significant difference in methylation status across the three disorders. So we used ANOVA model to do the hypothesis test, and the prior probability (*P* value) was corrected by Benjamini-Hochberg method for the control of the false discovery rate (FDR).

### 2.7. Analysis of Cytokine-Cytokine Interactome (CCI) and Hotspots

To investigate whether different AIDs had different CCI under DNA differential methylation status and identify the autoimmune inflammatory hotspots, the FEM package of R, of which the core algorithm was functional epigenetic modules (FEM), was performed based on the differential methylation status of key regions (e.g., TSS or 1st exon) and PPI [19]. In our analysis, the default parameters were used: the number of seeds was 100, the number of Monte Carlo runs was 1000, and the minimum number of molecules was 10. Finally, we set the threshold for significance to be less than 0.05.

### 2.8. Enrichment Analysis of Abnormal Methylated Gene Sets

Differential methylated sites or regions, which were located in the CpG islands and the neighbors, were annotated to coding genes, and then they were submitted to Gene Ontology (GO) enrichment analysis and Kyoto Encyclopedia of Genes and Genomes (KEGG) pathway analysis separately using R package “clusterProfiler” [20].

### 2.9. Statistical Software

The statistical analysis was performed by using R 3.4.1 (2017/06/30-Single Candle) and Bioconductor 3.5 on windows system x64.

## 3. Results

### 3.1. Differential Methylation Variabilities of Inflammatory Cytokines in SLE, RA, and pSS

The differential methylation variabilities (DMVs) are DNA methylation sites or regions with significant intragroup differences; DMVs includes differential methylated positions (DMPs) and differential methylation regions (DMRs). DMVs were the basic and most studied in DNA methylation studies. We conducted “dmpFinder” [13] and “DMRcate” [14] on the cytokine-relevant HumanMethylation beadchip (the abbreviation “Cytokine-Chip” will be used below) of the three AIDs to found DMVs, respectively.

In the Cytokine-Chip of SLE, we found 1219 significant DMPs that included 352 islands (28.88%), 323 shores (26.50%), and 82 shelves (6.73%). Of these DMPs, 671 (55.05%) showed higher methylation status, 492 (44.95%) were hypomethylated, and they corresponded to 492 genes (additional pkg). Moreover, 75 DMRs were found including 82 genes which are mainly located on chromosomes 6, 12, and 17, in which 33 DMRs were hypermethylated (44.0%) and 42 DMRs were hypomethylated (56.0%).

There were 6707 DMPs in patients with RA, covering 3662 islands and island-surrounding regions (54.60%), mapping to 689 genes and 2019 promoter-associated regions (30.10%). Compared with healthy controls, 4211 (62.79%) sites were significantly hypermethylated, and 495 (30.21%) were significantly hypomethylated. We found 64 DMRs including DMRs that were hypermethylated (48.4%) and 33 that were hypomethylated (51.6%).

In patients with pSS, 4716 DMPs were found in Cytokine-Chip, covering 2946 islands and island-surrounding regions (62.47%) and 1259 promoter-associated regions (26.70%), mapping to 670 genes. 1489 (31.57%) hypermethylated sites and 3227 (68.43%) hypomethylated sites were found in patients with pSS in comparison with health controls. In addition, 172 DMRs were revealed in patients with pSS including 217 genes with 24.4% (42/172) hypermethylated DMRs and 75.6% (130/175) hypomethylated DMRs, and the top 3 most frequently located chromosomes of DMRs were chromosomes 6, 11, and 19. In all, there are thousands of DMVs found in each of the three diseases, and the specific information of these DMVs is shown in Additional File 3 and the additional pkg. These results also indicated that abnormal methylation of inflammatory cytokine gene plays an important role in SLE, RA, and pSS.

### 3.2. Differences of DMVs in CpG Islands of Inflammatory Cytokines across SLE, RA, and pSS

Mammal CpG islands are basic sequences that are rich in CG two-nucleotide sequences and CpG dinucleotides, and the percentage of CG must be greater than 50% in this sequence. Canonical CpG islands have 300–3000 base pairs and have been found in approximately 70% of promoters located near the transcription start site (TSS) of a human gene such as housekeeping gene, tissue-specific gene, and regulator gene [21]. The methylation in CpG islands, as well as in CpG island-surrounding regions which contain shores and shelves, had strong correlation with the transcription initiation and chromosome configuration [22] and affected human health [23, 24]. Here, we defined the methylated differences across SLE, RA, and pSS with two principles: (i) there are statistical significances in the analyses of DMVs; (ii) in all three disorders, at least one disorder has a differential methylation status that is completely opposite to the other two, namely, if there is a significant hypermethylated site in SLE, then this site must be significantly decreased in the other two diseases. The delta-beta value (Δ*β*) was applied to assess the methylated differences in CpG islands across SLE, RA, and pSS, and the results were shown in Additional File 4.

**Figure 2:**
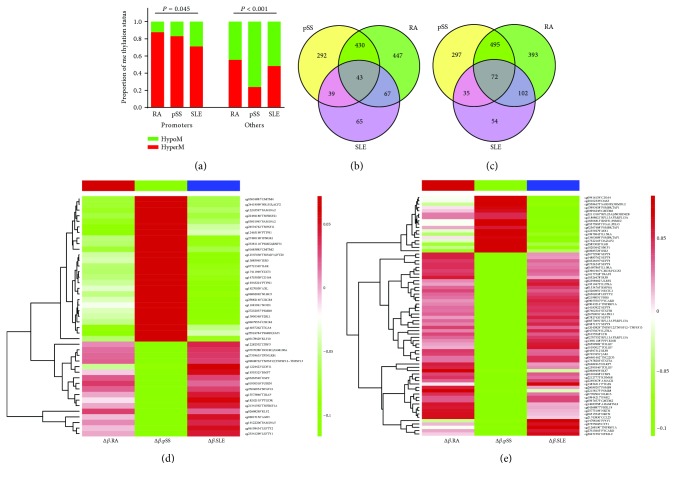
Identifying the differential methylation status of three AIDs in CpG islands and neighbor sites. (a) Distribution of methylation status in promoters and nonpromoters across SLE, RA, and pSS, and the proportion of that was calculated using Pearson's chi-squared test; (b-c) Venn diagrams of DMPs in the sites of CpGs and the neighbors (shores and shelves) common to the three AIDs separately; (d) heat map of 43 overlapping DMPs located in CpG islands; (e) heat map of 72 overlapping DMPs located in neighbors, and the detailed results of the two heat maps were in Additional File 4. RA: rheumatoid arthritis; SLE: systemic lupus erythematosus; pSS: primary Sjögren's syndrome.

On the basis of DMV analysis, we predicted the promoter region by using FANTOM project on the regions of CpG islands and the neighbors (namely, shores and shelves). The finding showed that the methylation status of DMPs in three AIDs was significantly different: the methylation status of promoter regions in the SLE was the lowest among the three AIDs, and the methylation status of nonpromoters in pSS was the lowest among the three AIDs (Figure 2(a)). It indicated that the methylated differences of the inflammatory cytokine genes did exist across SLE, RA, and pSS; thus, we further compared the CpG islands and the neighbors common to all DMVs across the three diseases (Figures 2(b) and 2(c)). Figures 2(b) and 2(c), respectively, depicted that the three AIDs shared 43 DMPs in CpG islands and 72 DMPs in the CpG island-surrounding regions. There were significant differences across SLE, RA, and pSS, such as *IL6R* (*P* = 5.65*E* − 79), *KLF10* (*P* = 2.69*E* − 75), *NR1H3* (*P* = 6.05*E* − 73), *CMTM4* (*P* = 1.69*E* − 68), *CD164* (*P* = 3.37*E* − 59), *TNFRSF21* (*P* = 3.63*E* − 51), and *STAT3* (*P* = 2.26*E* − 49). These 7 genes were all hypomethylated in SLE but opposite in pSS (Additional File 4). Not only that there are 10 methylated differences in the shared DMRs, including hypermethylated *CCR6*, *CMTM5*, *IL10RA*, *IL21R*, and *IL32* in SLE and pSS but also hypomethylated in RA. These outcomes indicated that methylation differences may be one of the reasons leading to different clinical manifestations and inflammatory damages of AIDs.

### 3.3. Similarities of DMVs of Inflammatory Cytokines across SLE, RA, and pSS

Abnormal methylations of cytokines gene in several key regions, such as TSS, CpG islands, and the neighbors, were likely to directly affect the transcription and gene function and contributed to the downstream signal network. We defined similarity and used it to describe DMVs with similar methylation status in three AIDs to find out the similarities of autoimmune inflammation across SLE, RA, and pSS. Here, we found 99 similarities of DMPs common to all three AIDs, and these DMPs corresponded to 86 inflammatory cytokine genes, of which the promoter of 32 genes had the same methylation status (Figure S3). These cytokine genes may be involved in important autoimmune processes in SLE, RA, and pSS, for example, *PIBF1* (3.48*E*−17) was involved in the formation of immune tolerance, but its promoter was hypermethylated in these three AIDs (Additional File 5).

**Figure 3:**
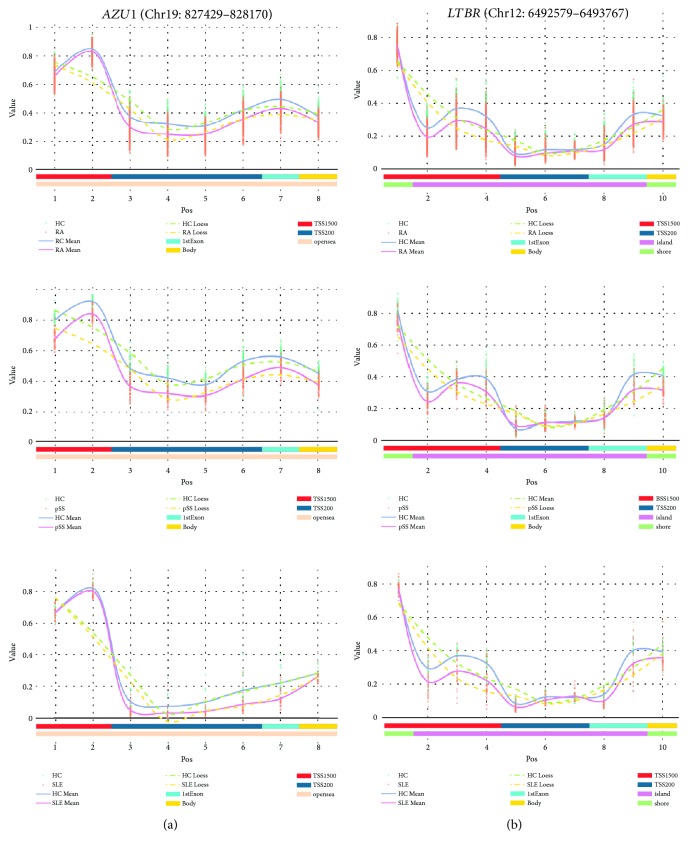
DMR curves that had similar methylation status among AIDs within the first exon and TSS200. Methylation for individual cases and controls is in orange and green, respectively. The solid lines in pink and gray separately stand for mean value of beta value of cases and controls. *x*-axis on bottom is not the actual MAPINFO, but the feature and CGI information of each DMP is plotted with various colors. (a) A DMR that starts at 827429 and 828170 to end located in *AZU1* within chromosome 19. (b) Curves of DMRs for *LTBR*. RA: rheumatoid arthritis; SLE: systemic lupus erythematosus; pSS: primary Sjögren's syndrome; HC: healthy controls; 1stExon: the first exon zone; TSS: the transcription start site; TSS1500: 1500 bp upstream of the TSS; TSS200: 200 bp upstream of the TSS.

Compared to DMPs, the DMRs had more important biological significance due to the similar methylation status in multiple consecutive CpG islands. In our study, we found 3 similarities of DMRs, which were *AZU1*, *LTBR*, and *RTEL1*. Importantly, the methylated segments of *AZU1* and *LTBR*, which are located in both TSS and the first exon zone, were all significantly hypomethylated (Figures 3(a) and 3(b)) and so did *RTEL1* (Figure S4). These similarities indicated that methylation loss of inflammatory cytokine genes might play important roles in the development of chronic inflammation or AIDs.

### 3.4. Cytokine-Cytokine Interactome (CCI) across SLE, RA, and pSS

Autoimmune inflammation was an interactome of various mediators. Coincidentally, the cytokine-cytokine interactome (CCI) was an important part of it and a part of protein-protein interaction (PPI) network used in FEM algorithm [19]. We hypothesized that abnormal methylation of cytokine genes was likely to involve in the imbalance or change of CCI. Therefore, we can use the FEM algorithm to investigate the differences of CCI in the three AIDs and identify inflammatory hotspots based on the methylated difference in diseases and PPI.

**Figure 4:**
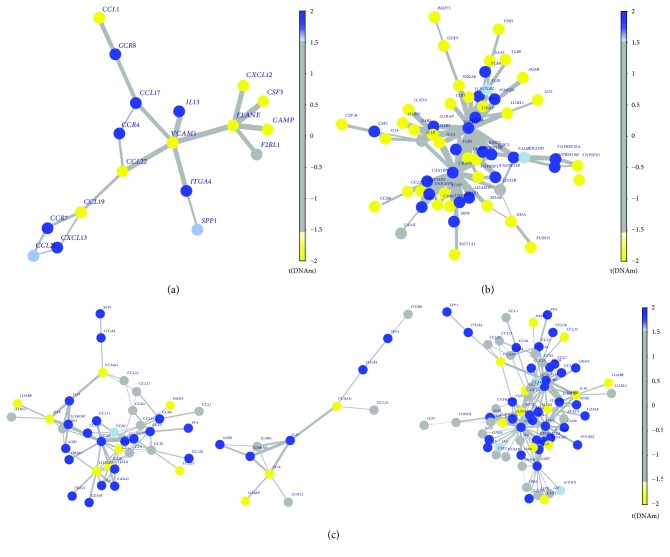
The hotspots of RA, pSS, and SLE. The yellow dot indicates genes annotated to hypomethylation, and the blue signaled hypermethylated genes to rich. (a) The hotspots centered around seed gene *VCAM1* and *ELANE* from RA dataset. (b) The hotspots *MYD88* calculated from dataset pSS. (c) Three CCI networks of SLE: hotspot *IL13*, *CCR5*, and *IFNG*, respectively. DNAm, changes of DNA methylation. Hotspots, the groups or modules calculated from FEM algorithm based on abnormal DNA methylation and protein interaction network.

In CCI analysis of RA, we identified two networks: one was 17 differential methylated genes centered around seed gene *VCAM1* (*P* = 0.016, Figure 4(a)), and the other one was 17 differential methylated genes, same to the former and contained *VCAM1*, centered around seed gene *ELANE* (*P* = 0.021). So the same interactome network was considered as and it suggested that the methylation loss of *VCAM1*, *ELANE*, and other 16 abnormal methylated genes was likely to play an epigenetic regulatory role in RA. There were 64 and 71 genes methylated abnormally within TSS200 or first exon centered around two seed genes (*MYD88* and *TRAF6*) in differential methylated interaction network of pSS, respectively. More than 80% of interacting members in the two diseases were also the same. And DNA methylation in hotspots (e.g., *MYD88*, *TRAF6*, *TICAM1*, and *MAVS*) was plotted in Figure 4(b). The detailed results were depicted in Supplementary Table S2 in Additional File 1. The CCI analysis of SLE also was performed in the meantime. *IL13* (*P* = 0.011), *CCR5* (*P* = 0.016), and *IFNG* (*P* = 0.011) were identified to act as hotspots, and most of interactive members in those CCI networks were hypermethylated (Figure 4(c)).

We summarize that the methylation loss of *VCAM1* and *ELANE* and hypermethylation of *MYD88* and *TRAF6* were vital in inflammation and immunity for RA and pSS, respectively. But compared with cytokine-cytokine interactome networks of RA and pSS, the network of SLE was more diverse and likely to indicate heterogeneity of SLE.

### 3.5. Similar Gene and Enrichment Analyses

**Figure 5:**
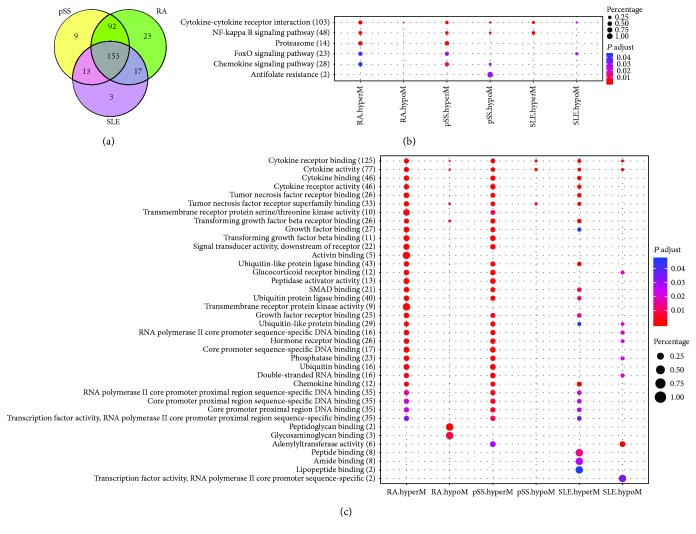
Comparison of GO and KEGG enrichment between hypo- and hypermethylated genes of different AIDs. The dot with red indicates high enrichment and the blue indicates low enrichment. The size of the dot represents the weight of this dot in each row, namely, the larger the point is, the more important the group is in the row. (a) Most of the abnormal methylated genes was overlapped. (b) The dot plot of KEGG pathway analysis. (c) The dot plot of the top 20 (most significant) GO terms of abnormal methylated cytokines. hyperM, hypermethylation; hypoM, hypomethylation.

In order to better understand how these abnormal cytokine genes affect biological effects, we first classified the cytokine gene according to the different methylation status and then conducted the Gene Ontology (GO) enrichment analysis and Kyoto Encyclopedia of Genes and Genomes (KEGG) pathway analysis and, finally, made a comparison on GO terms and KEGG pathways among SLE, RA, and pSS. After analyses, we found that there were 267 cytokine genes with abnormal methylated levels in their promoter regions in pSS compared to controls, 285 cytokine genes in RA, and 186 cytokine genes in SLE. However, more than half of these genes were mapped to CpG islands and the neighbors, and they had plenty of overlaps across the three AIDs (Figure 5(a)). Furthermore, we inputted these cytokine genes into R script clusterProfiler [20] for GO and KEGG pathway enrichment analyses. The significant biological processes (BP) and pathways were listed in Additional File 6. For visualization, the signal path enriched and the top 20 (ranked by adj. *P* value) terms were shown with dot plot (Figures 5(b) and 5(c)). We found that signal pathways “cytokine-cytokine receptor interaction,” “NF-kappa B signaling pathway,” “FoxO signaling pathway,” and “chemokine signaling pathway” were shared by RA, pSS, and SLE, and the top 10 GO terms in Figure 5(c) were related to “cytokine-cytokine interaction” and “NF-kappa B signaling pathway.” Interestingly, “glucocorticoid receptor binding” and “hormone receptor binding” were also enriched from hypomethylated cytokines in SLE, but they were enriched from hypermethylated genes in RA. Additionally, “glycosaminoglycan binding” was unique to hypomethylated genes of RA. The details of the pathway description had been shown in File 2 Table S3. The enrichment analyses suggested that the most inflammatory cytokine genes involved in AIDs were identical, but these same genes were likely to conduct a differential inflammatory process or signal pathway, and briefly, these abnormal methylated genes were essential for SLE, RA, or pSS to conduct inflammation or deteriorate disease and were good for targeted therapies.

## 4. Discussion

As high-throughput technology continues to evolve and the genome-wide DNA methylation profiling expands, we have been ushered into a new era, where massive biological data could be used for evidence-based research. In our study, we integrated multiple Cytokine-Chip from genome-wide DNA methylation datasets of SLE, RA, and pSS in order to find out the differences and similarities among these diseases. Taking the heterogeneity from different platform and demographic differences such as ethnicity of diverse datasets into account, the Illumina HumanMethylation450 BeadChip (450K Chip) and the Δ*β* of each probe between cases and controls were chosen for analysis. Particularly, the data from granulocytes, T, or B lymphocytes also combined for total inflammatory effects of peripheral blood leukocytes which consist of lymphocytes, monocytes, neutrophils, eosinophils, and so on.

Through a series of analyses depicted in Figure 1, we identified 6707 DMPs and 64 DMRs in RA, 4716 DMPs and 172 DMRs in pSS, and 1219 DMPs and 75 DMRs in SLE. Moreover, the methylation status of promoter regions in the SLE was the lowest among the three AIDs and the methylation status of nonpromoters in pSS was the lowest (Figure 2(a)). Importantly, the methylation statuses of 43 overlapping DMPs in CpG islands and 72 DMPs in the CpG island-surrounding regions were significantly different across the three AIDs and many vital proinflammatory genes were included [25–28] such as *IL6R*, *IFNGR1*, *STAT3*, *PSMB9*, *PSMB8*, *TNFRSF12*, *TNFRSF1A*, *TNFSF12*-*TNFSF13*, *CD164*, and *TRAF5* (Figures 2(d) and 2(e)). Not only that there are 10 methylated differences in the shared DMRs, such as *CCR6*, *CMTM5*, *IL10RA*, *IL21R*, and *IL32*, were all hypermethylated in SLE and pSS but also hypomethylated in RA (Additional File 4). It indicated that aberrant DNA methylation did occur in various autoimmune diseases [4, 29], and many of them were particular to each disorder except for some shared ones, namely, methylation differences of cytokines across various AIDs may be one of reasons leading to different clinical manifestations and inflammatory damages.

Meantime, we did find out several commonness of autoimmune inflammation across SLE, RA, and pSS: the three AIDs had 99 DMPs (Figure S3) and 3 DMRs with similar methylation status (Additional File 5). Since these DMVs are located in the CpG islands and the neighbors, the cytokines of these DMVs were likely to represent similar inflammatory signals or functions in all three AIDs. For instance, *PIBF1* (cg12930920), a progesterone immunomodulatory binding factor whose promoter was hypermethylated in all three AIDs, is probably involved in the formation of immune tolerance and maintenance of pregnancy. Therefore, the three more important DMRs (*AZU1*, *LTBR*, and *RTEL1*) attracted our attention. *AZU1*, *LTBR*, and *RTEL1* were characterized by DNA hypomethylation in TSS or first exon regions among SLE, pSS, and RA. *AZU1* encodes azurocidin, proteinase 3, and neutrophil elastase in a cluster located at the short arm of human chromosome 19 (19p13.3). All 3 proteins contribute to innate immune response by destroying microorganism. Azurocidin was also involved in monocyte recruitment in inflammatory. Lee et al. also highlighted that azurocidin can upregulate the expression of *VCAM1*, *ICAM1*, and selectin to enhance adhesion of inflammatory cells [30]. In addition, it is well known that the autoantibodies against proteinase 3 (PR3) acted as an obligate feature in developing systemic autoimmune vasculitis such as Wegener's granulomatosis [31]. Interestingly, compared to subjects without steroid use, a methylation loss of *AZU1* was showed in the ones with steroid usage [32].

Lymphotoxin beta receptor (LT*β*R), a member of the tumor necrosis factor receptor superfamily, was reported to be associated with chronic inflammation diseases such as viral encephalitis [33], hepatitis B [34], IgA nephropathy [35], lymphoblastic leukemia [36], and type 1 diabetes [37] and plays a critical role in immune response and initiation of inflammation. Moreover, LT*β*R signaling is likely to involve in the activation of NF*κ*B [38], the regulation of type I interferon axis in dendritic cells and CD8^+^ T cell [39], and the induction of TLR cross-tolerance [40]. Fava et al. proposed that the ligation of LT*β*R could reduce the loss of salivary secretion rates and improved ocular surface integrity score in NOD mouse model of Sjögren's syndrome [41]. Recently, disturbance of lymphotoxin/LIGHT signaling axis in Sjögren's syndrome also be announced [42]. Therefore, *LTBR* hypomethylation not only plays a pivotal role in the ignition of autoimmunity but also is a potential therapeutic target.

In addition, *RTEL1*, encoding for the regulator of telomere elongation helicase 1, is involved into telomere-length regulation, DNA repair, and genomic stabilization. More studies brought up that the mutation of *RTEL1* had been linked to dyskeratosis congenital [43], Hoyeraal-Hreidarsson syndrome [44], pulmonary fibrosis [45], myelodysplastic syndrome [46], and lung cancer [47] and even rheumatoid arthritis-associated interstitial lung disease [48]. Although mechanism of how the mutation of *RTEL1* participated in fibrosis and dysfunction of immune system is unclear, the untested hypomethylation in this gene should be given enough attention.

As we have illustrated, 3 hypomethylated genes involved in RA, pSS, and SLE could serve as initiators in the autoimmunity and inflammatory process. However, the autoimmunity and inflammation require a lot of inflammatory mediators such as interleukin, lymphokine, or chemokine to form a network in which mediator orchestrates proinflammatory cascades [49]; hence, we performed CCI analysis [19] based mainly on changes of DNA methylation in TSS200, first exon or TSS1500, protein-protein network [19, 50], and the interesting phenotype to identify this inflammatory network due to differential methylation of cytokine gene (also named as “hotspots”). Five hotspots were identified as biologically plausible pathways in inducing or perpetuating of inflammation which are epigenetically deregulated in AIDs.

In these hotspots, not only the landscape of Toll-like receptor- (TLR-) induced pathways, interferon (IFN) signature, and chemokine/adhesion molecule signaling were presented but also the imbalance of activated T cell and regulatory T cell was revealed. Myeloid differentiation factor 88 (MyD88) is the key members of TLR pathway, and it transfers the antigen signature to trigger a series of signaling cascades that culminate in transcription of numerous downstream genes such as inflammatory cytokines, chemokines, interferons, lymphokine, and complement factors [51, 52]. The overactivation of TLR resulting in perpetuation of inflammation in autoimmunity has been confirmed by numerous studies. In addition, hydroxychloroquine has been recommended to effectively treat pSS and SLE due to its suppression to TLR7 to attenuate inflammation [53]. Thereby, the methylation change of MyD88, TRAF6, and other surrounding molecules may play a role in the pathogenesis of pSS in parallel with other supporting proinflammatory network (Figure 4(b)). The type I IFN system also activates in many autoimmune disorders particularly SLE [52, 54], and the epigenetic susceptibility of interferon-regulated genes in SLE has been reported by several genome-wide DNA methylation studies [9, 10, 55].

These inflammatory signaling pathways do not simply stand alone or act independently; a robust evidence is that TLR, retinoic acid-inducible gene I-like receptors (RLR), nucleotide oligomerization domain-like receptors (NLR), and INF act together to promote inflammation, but it is clearly a relationship of checks and balances among them [51, 53]. This relationship can also be observed between cells and cells as exemplified by the imbalance of Th17/Treg in SLE patients [52, 56, 57], and this imbalance is also indirectly observed in our hotspots: the *CCR5* encoding a membrane molecule in Foxp3+ Treg was hypermethylated, but the demethylated CCL2 was able to enhance a systemic immune response in proinflammatory cells [58] (Figure 4(c)). It is remarkable that *AZU1* and *VCAM1* (Figure 4) hypomethylated in their genes presumably combine their partners (e.g., chemokines and adhesion receptors) to trigger vasculitis that can be observed in most of autoimmune conditions including RA and SLE [30, 59, 60].

Our enrichment analysis had classified these aberrant methylated genes as “cytokine-cytokine receptor interaction,” “NF-kappa B signaling pathway,” “FoxO signaling pathway,” and other signaling pathways, but only the aberrant genes of RA and pSS appeared in the proteasome signaling system (Figure 5(b)). More importantly, the cluster of hypomethylated cytokines of pSS assembled in “antifolate resistance” to promote inflammation, and it is consistent with the clinical observation: most RA patients with arthritis prescribed methotrexate is effective, but the pSS patients has no effect. In our future studies, the results will be further validated.

DNA methylation of some candidate genes has been developed as epigenetic biomarkers for diagnoses in cancer such as bladder cancer [61], breast cancer [62], and cholangiocarcinoma [63]. Moreover, antineoplastic agents 5-azacytidine, 5-deoxycytidine and zebularine were DNA methyltransferase (DNMT) inhibitors. They can block DNMT during DNA synthesis resulting in DNA demethylation in promoter regions of the tumor suppressor genes. Based on our results, the treatment of AIDs should promote DNA methylation of inflammatory cytokines rather than promoting demethylation like antitumor therapies. In particular, the degree of demethylation in promoter regions in SLE was the most severe (Figure 2(e)) and we could perhaps utilize the antisense oligonucleotide inhibitors and S-adenosylmethionine to treat SLE by recovering the hypomethylation of inflammatory cytokines in future studies.

There are subclinical immunological processes before the presence of AIDs. As elaborated by recent studies, if we illustrated the course of AIDs as a river, the subclinical immunological and undifferentiated status of AIDs acts as the upstream of the river in AID development; as the disease progresses, small streams can branch out (different clinical symptoms) and meet formal classification criteria for SLE, RA, and pSS, respectively [64]. In our study, we showed the specific similarities (perhaps the upstream of the river) of the three classic representatives of AIDs, and it indicated that targeting the similarities perhaps could improve the treatment or early diagnosis of AIDs.

## 5. Conclusion

In summary, we systematically profiled the DNA-methylated patterns for cytokine genes in SLE, RA, and pSS using human methylation microarray. Many similarities and differences of DMVs of inflammatory cytokines across three AIDs were identified. The epigenetic susceptible candidates including *AZU1*, *LTBR*, *RTEL1*, and *VCAM1* were identified as triggers of autoimmune signaling cascades and contributed to derailed pro-/anti-inflammatory cells. The disturbance of TLR signaling in pSS and the overactivation of type I IFN system in SLE owed to aberrant DNA methylation were confirmed, and *AZU1* and *VCAM1* were presumably involved in the pathogenesis of vasculitis. The epigenetic candidate genes may be the potential biomarkers or therapeutic targets of AIDs. Systemic multicenter genetic studies including epigenome, transcriptomics, whole genomic sequencing, and prospective studies are needed to reveal underlying causes of epigenetic changes and preclinical discoveries. Additionally, the robust bioinformatic analysis cross genomics research and the evidence-based algorithm are suggested to be developed.

## Data Availability

All datasets generated as part of this study are available at https://www.ncbi.nlm.nih.gov/gds/ under the following accession numbers GSE42861, GSE75679, GSE59250, GSE65097, and GSE82218.

## Supplementary Materials

Supplementary 1 Additional File 1: a document (.docx) contains all supplementary figures and tables.

Supplementary 2 Additional File 2: summary of Cytokine-Chip (.xlsx). The information of probes and the annotation of gene in Cytokine-Chip were described in this file.

Supplementary 3 Additional File 3: the DMVs of each disease were listed in this document (.xlsx).

Supplementary 4 Additional File 4: the differences of DMVs in CpG islands across the three AIDs were listed in this document (.xlsx).

Supplementary 5 Additional File 5: the similarities of DMVs across the three AIDs were listed in this document (.xlsx).

Supplementary 6 Additional File 6: the results of Gene Ontology enrichment analysis and KEGG pathway analysis were listed in this document (.xlsx). Additional pkg: the results of meta-analysis of dataset SLE. slemeta_results.xlsx described all the results of meta-analysis; slemeta_forest plot.pdf contained 212 forest plots that been drawn according to the DMPs. probe to forest.xlsx annotated the forest plot.
