# Supplementary material for: A DNA-Methylated Sight on Autoimmune Inflammation Network across RA, pSS, and SLE
Source: J Immunol Res. 2018 Aug 12;2018:4390789. doi: 10.1155/2018/4390789 (PMC6109517; doi:10.1155/2018/4390789)
Supplement: Supplementary 1 — Additional File 1: a document (.docx) contains all supplementary figures and tables. [file 4390789.f1.docx]

| Table S1 Characteristics of datasets enrolled and basic demographics. | | | | | |
| --- | --- | --- | --- | --- | --- |
| Characteristics | Samples | | | | |
| Accession number | GSE42861 | GSE75679 | GSE59250 | GSE65097 | GSE82218 |
| PMID | 23334450 | 26966136 | 23950730 | 25638528 | 27412348 |
| Sample size | 689 | 48 | 434 | 38 | 55 |
| Year | 2013 | 2015 | 2014 | 2015 | 2016 |
| Platform | GPL13534 | | | | |
| Method | Illumina HumanMethylation450 BeadChip | | | | |
| Population | Swedish | Norwegians | NR | American | NR |
| Country | Sweden | Sweden | USA | USA | USA |
| Sample source | PBLs | PBLs | PBLs | Neutrophils+ LDG | PBMC |
| Cases | RA | pSS | SLE | SLE | SLE |
| Gender | | | | | |
| Female | 492 | 41 | NR | 38 | 46 |
| Male | 197 | 7 | NR | 0 | 9 |
| Age | | | | | |
| Mean±SD(years) | 51.93±11.80 | 56.54±13.72 | NR | 37.84±9.94 | 30.49±8.63 |
| Abbreviations: PBLs, peripheral blood leukocytes; LDG, low-density granulocytes; PBMC, peripheral blood mononuclear cells, NR, not reported; SD, standard deviation. | | | | | |


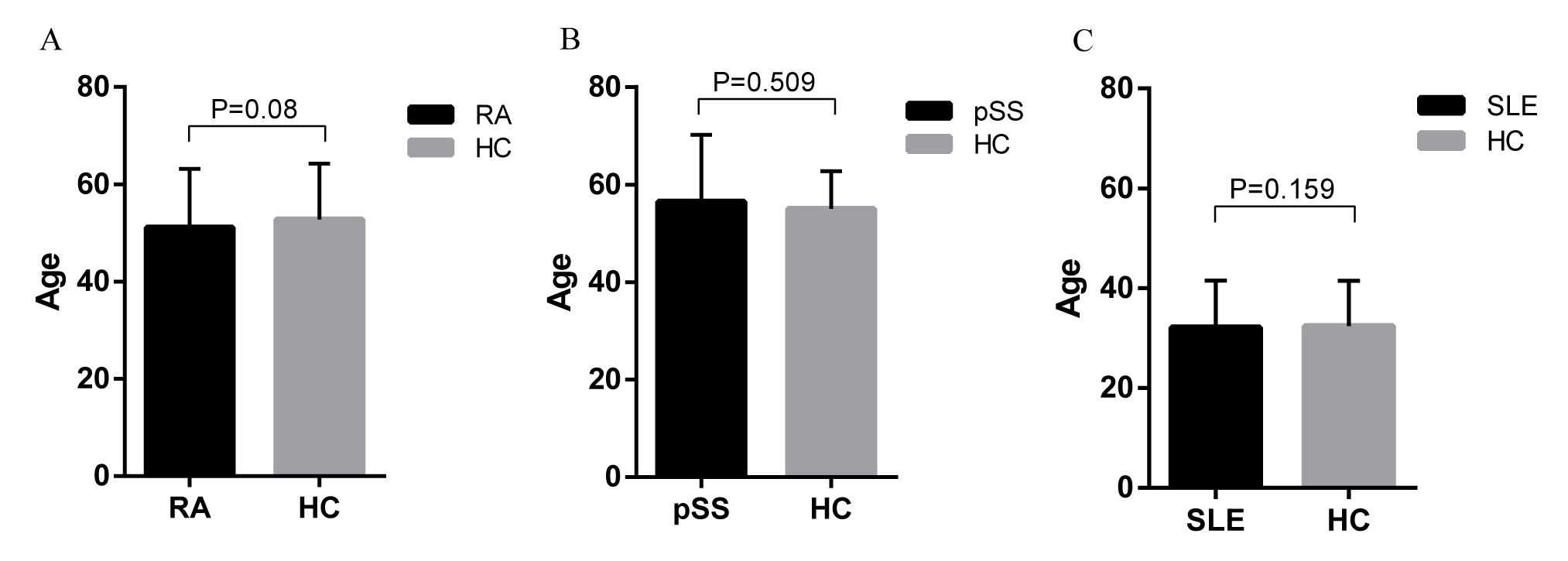


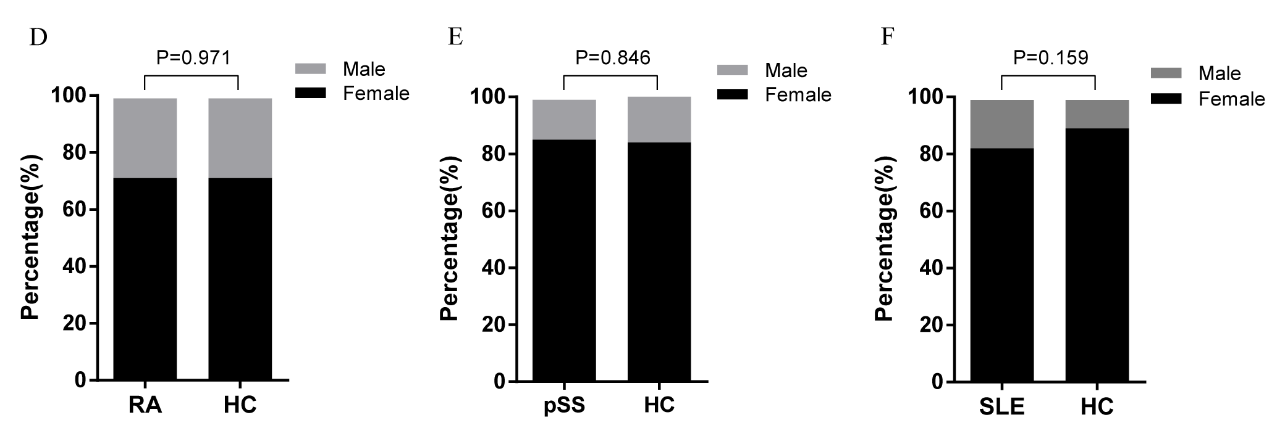


**Figure S1** Difference of gender and age between cases and controls. (A-C) Histograms show that it is no significant difference in age of patients compared to controls, as well as in sex.(D-F)Percentage bar diagram of gender rheumatic patients and controls.


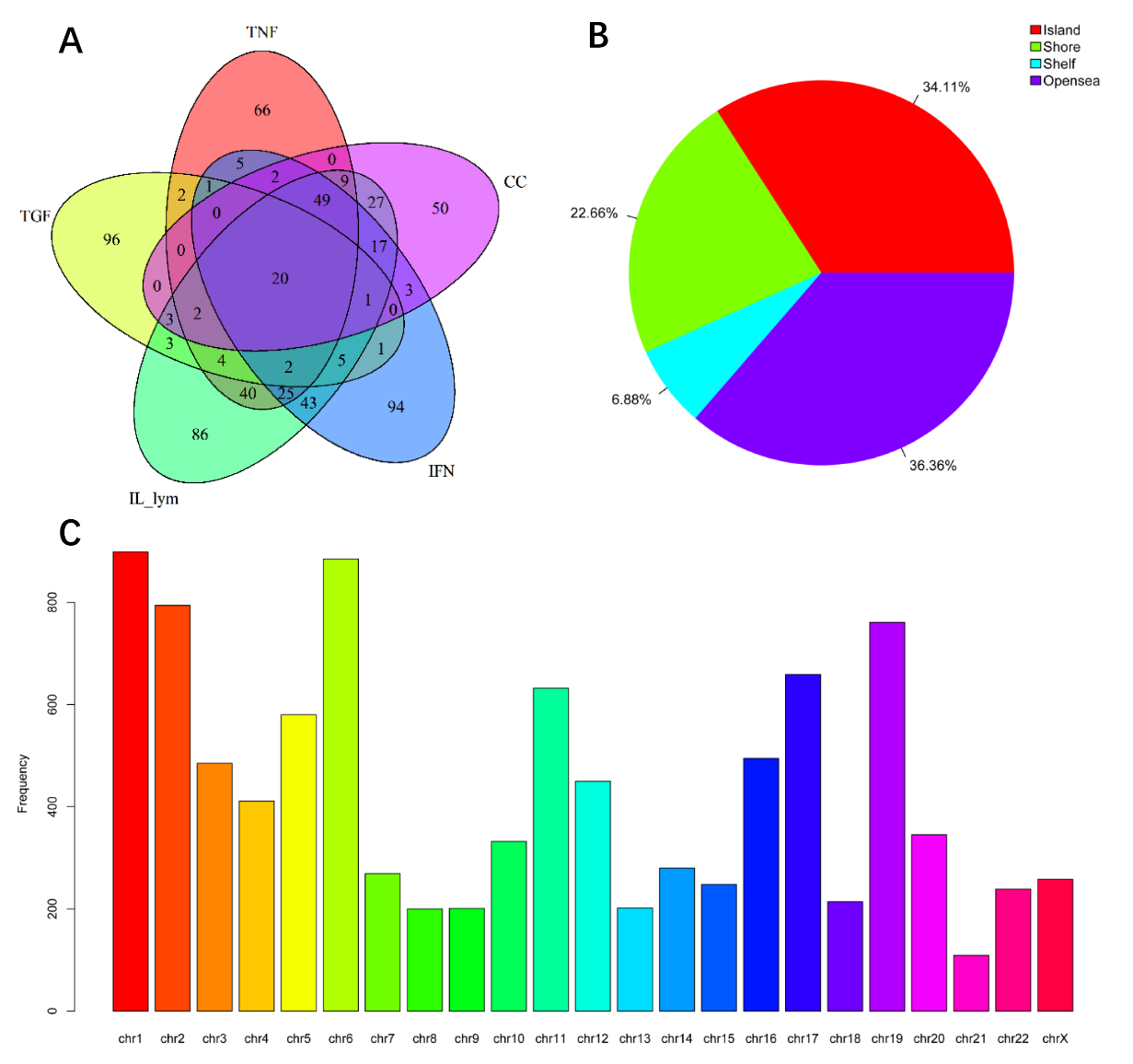


**Figure S2** Characteristic of Cytokine Relevant HumanMethylation beadchip. (A) Venn diagram of six kinds of cytokine relevant genes. (B, C) Histogram of cytokine relevant probes to mapped to genome. Abbreviations: TNF, Tumor Necrosis Factors superfamily; CC, Chemokines; IFN, Interferons; IL_Lym, Interleukins and Lymphokines; TGF, Transforming Growth Factor; chr, chromosome.


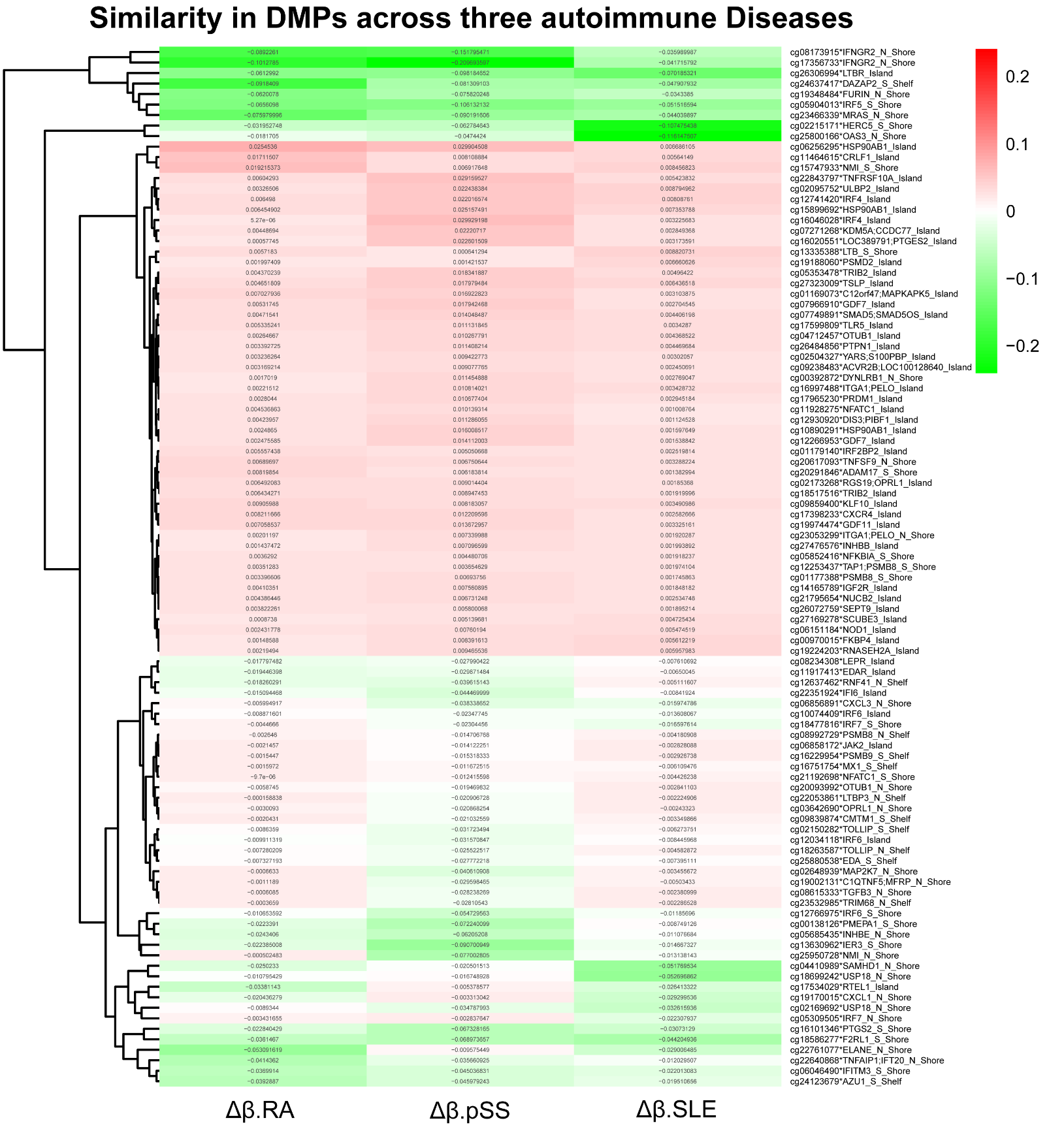


**Figure S3** The heatmap of similar methylation status in DMPs across SLE, RA and pSS. The blue to the red represent the increasing level of DNA methylation, and the numbers in the cell is the Δβ. The annotations text on the right consist of probe names, gene ID and information of CpG islands.


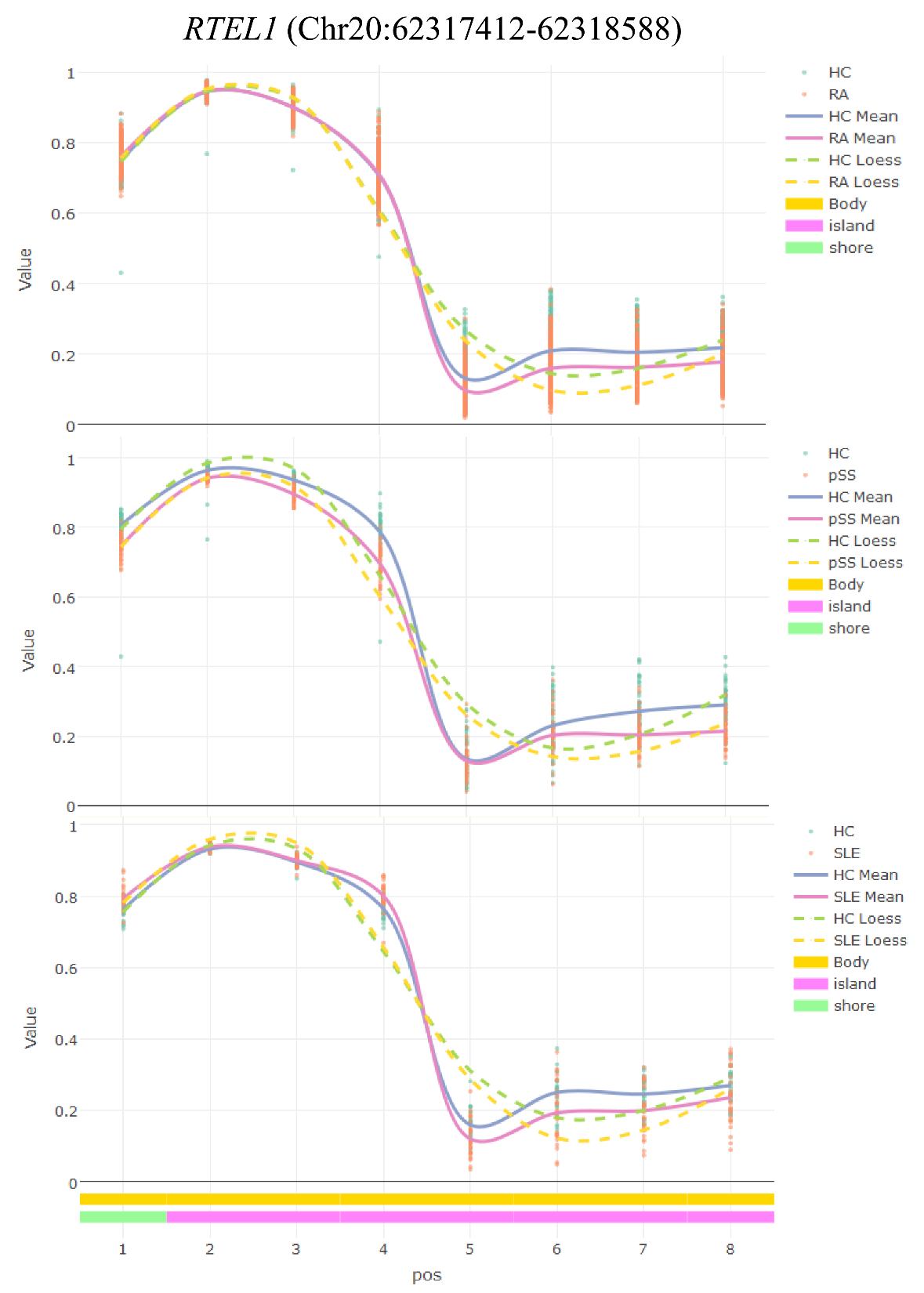


**Figure S4** Curves of abnormal methylation in segment of *RTEL1.* It was hypomethylated in three AIDs but located in gene body not in TSS or 1stExon. Abbreviations: RA: rheumatoid arthritis; SLE: systemic lupus erythematosus; pSS: primary Sjögren syndrome; HC: healthy controls; 1stExon: the first exon zone.

Table S2 Results of Differential Cytokines-Cytokines Interactome by FEM analysis

| **disease** | **Seed.gene** | **Size** | **Mod** | **Pvalue** | **Genes** |
| --- | --- | --- | --- | --- | --- |
| **pSS** | MYD88 | 65 | 6.992516 | 0.001 | MYD88 IL1B S100A8 TRAF6 BIRC3 FADD TLR2 IL1RAP BIRC2 TICAM1 TNFRSF13B IRF4 TRAF5 HMGB1 BGN TIRAP TLR1 TOLLIP TLR3 IL1R1 IRF5 IL1A TLR5 TLR7 IL1RL1 MAVS IL1R2 IL1RN TNFRSF17 TNFSF13B ADAM17 IL1F10 EDA2R SPHK1 XIAP AGER CD40 CCL20 TRAF3IP2 EDAR TLR9 TNFAIP3 TAX1BP1 TNFSF11 CHUK CRADD TLR6 CCR6 SAA1 FPR1 IL33 RIPK3 PRDM1 TANK GDF9 BMP15 DAPK1 IL16 CSF1 CSF1R TNFRSF10A TNFRSF10B TNFSF10 EDA FURIN |
|  | TRAF6 | 72 | 6.987689 | 0 | TRAF6 IL1B CHUK BIRC3 NFKBIA NFKB1 TRAF3IP2 TNFAIP3 TNFSF11 CD40 XIAP MYD88 BIRC2 ADAM17 TICAM1 IRF8 EDAR SPHK1 TNFRSF13B TNFRSF17 TRAF5 TIRAP TOLLIP MAVS TLR3 IL1R1 EDA2R IRF5 IL1A CCL20 TAX1BP1 TNFSF13B REL IL1RAP IL1R2 IL1RN S100A8 IL1RL1 IL1F10 BCL2A1 TLR2 HMGB1 AGER TLR7 FADD BGN TLR1 TLR5 TLR9 SLC11A1 IRF2 IRF2BP2 NFKB2 USP2 CRADD TLR6 CCR6 IL33 SAA1 FPR1 RIPK3 TANK DAPK1 CEBPB GDF9 BMP15 IL16 CSF1 CSF1R LTBR TNFSF14 IRF1 |
| **RA** | ELANE | 18 | 7.164076 | 0.021 | ELANE VCAM1 CXCL12 F2RL1 CSF3 CAMP ITGA4 CCL22 CCL17 CCR4 SPP1 CCL19 CCR7 CCL21 CXCL13 IL13 CCR8 CCL1 |
|  | VCAM1 | 18 | 7.164076 | 0.016 | VCAM1 ITGA4 ELANE CCL17 CCL22 IL13 CAMP CSF3 F2RL1 CXCL12 CCR4 SPP1 CCL19 CCR7 CCL21 CXCL13 CCR8 CCL1 |
| **SLE** | IL13 | 12 | 3.208739 | 0.011 | IL13 IL18R1 IL18RAP VCAM1 IL18 NOS2 IL18BP IL1RL2 ITGA4 SPP1 CCL22 ITGB6 |
|  | IFNG | 73 | 2.736263 | 0.011 | IFNG IFNGR1 IL2 JAK2 TNF IL18R1 IL18RAP IL12RB2 IL27RA IL12B IL23A CD28 IL18 IFNGR2 EBI3 IL6ST IL27 IL12A IFNAR2 FASLG IL13 IL19 CXCL1 IL4 CCL3 NOS2 IL6 IL6R IL24 IL20RA CCL4 IL18BP VCAM1 CCL2 CCR5 CCL11 CCR2 CCL13 CCL8 CCL7 ORM1 IL3 IL1RL2 IL11RA CSF2 OSMR OSM GZMB IL31RA LIFR CTF1 LIF IL5 IL5RA STAT4 PTAFR IL20 ITGA4 SPP1 EPO TNFRSF1A CCL22 CCR4 CCL5 CCR1 PF4 CCL23 CCL26 CCL16 HRH4 CCR8 CCL17 CCL1 |
|  | IL18RAP | 12 | 3.208739 | 0.025 | IL18RAP NOS2 IL18R1 IL18 IL13 IL18BP IL1RL2 VCAM1 ITGA4 SPP1 CCL22 ITGB6 |
|  | CCR5 | 39 | 2.714644 | 0.016 | CCR5 IL18R1 IL18RAP IL12RB2 CCR2 CCL8 CCL7 CCL2 CCL13 IL12B ORM1 IL18 IL12A CCL3 CCL4 CCL5 CCL11 CCL16 IL13 FASLG CCR1 NOS2 IL4 CCR4 PF4 IL18BP CCL23 CCR8 CCL1 CCL17 VCAM1 CCL22 CCL26 IL1RL2 GZMB HRH4 ITGA4 SPP1 TBX21 |
|  | IL18 | 12 | 3.208739 | 0.01 | IL18 IL18R1 IL18RAP IL13 IL18BP IL1RL2 NOS2 VCAM1 ITGA4 SPP1 CCL22 ITGB6 |
|  | NOS2 | 12 | 3.208739 | 0.016 | NOS2 IL18R1 IL18RAP IL13 IL18 IL18BP IL1RL2 VCAM1 ITGA4 SPP1 CCL22 ITGB6 |
|  | JAK2 | 65 | 2.720107 | 0.018 | JAK2 IL1B IFNGR1 IL2 IRS2 PLCG2 TNFRSF1A TNF IL12RB2 CCR5 MPL PTPRC IL27RA IL4 IL6 CXCL12 CCR2 CCL2 IL12B IL23A EPO CSF2 IL5 IL5RA FASLG IL3 IFNGR2 IFNG IL6R EBI3 IL6ST IL27 IL12A PTAFR CCL3 CXCL1 CCL4 IL19 OSMR IL24 CD28 CCL11 CCL13 CCL8 CCL7 IL20RA IFNAR2 IFNAR1 ORM1 IL16 CSF1 IL11RA TEC OSM GZMB IL31RA LIFR CTF1 LIF IL20 CCL16 HRH4 CCR8 CCL1 CCL17 |
|  | PPBP | 11 | 1.655718 | 0.034 | PPBP ALB HP PF4V1 CXCR2 CD163 CXCL2 ORM1 AMBP CXCL5 CXCL3 |
|  | HP | 11 | 1.655718 | 0.044 | HP ALB PPBP PF4V1 CD163 ORM1 CXCR2 CXCL2 AMBP CXCL5 CXCL3 |
|  | CCR2 | 21 | 1.443324 | 0.048 | CCR2 CCR5 CCL8 CCL7 CCL2 CCL13 CCL11 CCL16 HRH4 CCR1 CCL5 CCL23 ORM1 PF4 CCL26 CCR4 CCL3 CCL4 CCR8 CCL1 CCL17 |
|  | CD163 | 11 | 1.655718 | 0.048 | CD163 HP PPBP PF4V1 ALB ORM1 CXCR2 CXCL2 AMBP CXCL5 CXCL3 |
| Abbreviations: RA: rheumatoid arthritis; SLE: systemic lupus erythematosus; pSS: primary Sjögren syndrome. | | | | | |

Table S3 The descriptions of KEGG pathway enriched in our study.

| **ID** | **pathway name** | **function description** |
| --- | --- | --- |
| map03050 | Proteasome | The proteasome is a protein-destroying apparatus involved in many essential cellular functions, such as regulation of cell cycle, cell differentiation, signal transduction pathways, antigen processing for appropriate immune responses, stress signaling, inflammatory responses, and apoptosis. It is capable of degrading a variety of cellular proteins in a rapid and timely fashion and most substrate proteins are modified by ubiquitin before their degradation by the proteasome. The proteasome is a large protein complex consisting of a proteolytic core called the 20S particle and ancillary factors that regulate its activity in various ways. The most common form is the 26S proteasome containing one 20S core particle and two 19S regulatory particles that enable the proteasome to degrade ubiquitinated proteins by an ATP-dependent mechanism. Another form is the immunoproteasome containing two 11S regulatory particles, PA28 alpha and PA28 beta, which are induced by interferon gamma under the conditions of intensified immune response. Other regulatory particles include PA28 gamma and PA200. Although PA28 gamma also belongs to a family of activators of the 20S proteasome, it is localized within the nucleus and forms a homoheptamer. PA28 gamma has been implicated in the regulation of cell cycle progression and apoptosis. PA200 has been identified as a large nuclear protein that stimulates proteasomal hydrolysis of peptides. |
| map01523 | Antifolate resistance | Since the 1940s, antifolates have played a pivotal role in drug treatment of malignant, microbial, parasitic and chronic inflammatory diseases. The molecular basis of the anti-proliferative activity of antifolates relies on inhibition of key enzymes in folate metabolism, which results in disruption of purine and thymidylate biosynthesis, inhibition of DNA replication and cell death. The anti-inflammatory properties of antifolate have been most strongly related to its ability to block the release of pro-inflammatory cytokines such as tumor necrosis factor (TNF)-alpha or interleukin (IL)-1beta. Cells may develop resistance to an antifolate drug by virtue of impaired drug transport into cells, augmented drug export, impaired activation of antifolates through polyglutamylation, augmented hydrolysis of antifolate polyglutamates, increased expression and mutation of target enzymes, and the augmentation of cellular tetrahydrofolate-cofactor pools in cells. |
| map04060 | Cytokine-cytokine receptor interaction | Cytokines are soluble extracellular proteins or glycoproteins that are crucial intercellular regulators and mobilizers of cells engaged in innate as well as adaptive inflammatory host defenses, cell growth, differentiation, cell death, angiogenesis, and development and repair processes aimed at the restoration of homeostasis. Cytokines are released by various cells in the body, usually in response to an activating stimulus, and they induce responses through binding to specific receptors on the cell surface of target cells. Cytokines can be grouped by structure into different families and their receptors can likewise be grouped. |
| map04064 | NF-kappa B signaling pathway | Nuclear factor-kappa B (NF-kappa B) is the generic name of a family of transcription factors that function as dimers and regulate genes involved in immunity, inflammation and cell survival. There are several pathways leading to NF-kappa B-activation. The canonical pathway is induced by tumour necrosis factor-alpha (TNF-alpha), interleukin-1 (IL-1) or byproducts of bacterial and viral infections. This pathway relies on IKK- mediated IkappaB-alpha phosphorylation on Ser32 and 36, leading to its  degradation, which allows the p50/p65 NF-kappa B dimer to enter the nucleus and activate gene transcription. Atypical pathways are IKK-independent and rely on phosphorylation of IkappaB-alpha on Tyr42 or on Ser residues in IkappaB-alpha PEST domain. The non-canonical pathway is triggered by particular members of the TNFR superfamily, such as lymphotoxin-beta (LT-beta) or BAFF. It involves NIK and IKK-alpha-mediated p100 phosphorylation and processing to p52, resulting in nuclear translocation of p52/RelB heterodimers. |
| map04068 | FoxO signaling pathway | The forkhead box O (FOXO) family of transcription factors regulates the expression of genes in cellular physiological events including apoptosis, cell-cycle control, glucose metabolism, oxidative stress resistance, and longevity. A central regulatory mechanism of FOXO proteins is phosphorylation by the serine-threonine kinase Akt/protein kinase B (Akt/PKB), downstream of phosphatidylinositol 3-kinase (PI3K), in response to insulin or several growth factors. Phosphorylation at three conserved residues results in the export of FOXO proteins from the nucleus to the cytoplasm, thereby decreasing expression of FOXO target genes. In contrast, the stress-activated c-Jun N-terminal kinase (JNK) and the energy sensing AMP-activated protein kinase (AMPK), upon oxidative and nutrient stress stimuli phosphorylate and activate FoxOs. Aside from PKB, JNK and AMPK, FOXOs are regulated by multiple players through several post-translational modifications, including phosphorylation, but also acetylation, methylation and ubiquitylation. |
| map04062 | Chemokine signaling pathway | Inflammatory immune response requires the recruitment of leukocytes to the site of inflammation upon foreign insult. Chemokines are small chemoattractant peptides that provide directional cues for the cell trafficking and thus are vital for protective host response. In addition, chemokines regulate plethora of biological processes of hematopoietic cells to lead cellular activation, differentiation and survival.  The chemokine signal is transduced by chemokine receptors (G-protein coupled receptors) expressed on the immune cells. After receptor activation, the alpha- and beta-gamma-subunits of G protein dissociate to activate diverse downstream pathways resulting in cellular polarization and actin reorganization. Various members of small GTPases are involved in this process. Induction of nitric oxide and production of reactive oxygen species are as well regulated by chemokine signal via calcium mobilization and diacylglycerol production. |
